# Improving accessebility to psychiatry in NHS Tayside

**DOI:** 10.1192/bjo.2021.546

**Published:** 2021-06-18

**Authors:** Thomas Leung, Lori-an Etherington, Neil Stevenson

**Affiliations:** NHS Tayside

## Abstract

**Aims:**

Our aim is to improve the accessibility of Psychiatry to other specialties when being contacted for review and advice, both in hours and out of hours.

**Background:**

From clinical contact and informal conversations, other specialties sometimes have difficulties contacting psychiatry for advice/review. The aim of this is quality improvement project is to determine how accessible we are to other specialties and work on improving how we communicate with the general hospital.

**Method:**

We created a questionnaire for colleagues from other specialties to fill in from 26/9/19 for 6 weeks. We gathered information regarding their grade, work site, previous contact with psychiatry, whether they knew where to find our contact information and if they could identify the correct method to ask for advice from general adult psychiatry (GAP), Psychiatry of old age (POA) , and out of hours psychiatry (OOH). We also asked colleagues to put in free text comments regarding their experience in contacting psychiatry. We also asked if our colleagues were aware of how to perform an Emergency Detention Certificate as this is advice we sometimes give which does not always need our input immediately.

**Result:**

There was a total of 39 responses, 29 from Ninewells Hospital (NW) and 10 from Perth Royal Infirmary (PRI). There was a mixture of staff grades from Foundation Doctors to Consultants. 23/39 colleagues knew where to find contact information for Psychiatry, 14/39 colleagues correctly answered how to contact GAP (Phone), 15/39 colleagues correctly answered how to contact POA (Email), 15/39 colleagues correctly identified who to contact OOH, and 16/34 colleagues who could do emergency detentions (FY2+) knew how to do one. Free text comments often referred back to the difficulty of finding the right grade of staff first try, Feedback from PRI where there was no dedicated Liaison Service and relies on a duty doctor system was less positive, with terms ‘tricky’, ‘difficulty’, ‘awkward’ used in majority of responses.

**Conclusion:**

From our results we can conclude that contacting Psychiatry in NHS Tayside can be confusing for other specialties. Taking this forward, we will utilize the ‘referral finder’ system in NHS Tayside and review the existing information available, and to update the contact information for our subspecialties to make contact ourselves more streamlined and accessible. We will also review appropriate clinical protocols that we can link to our page on referral finder to help save time for our colleagues as well.

